# Identification and Characterization of *Fusarium incarnatum* Causing Leaf Spot and Fruit Rot on Luffa in China

**DOI:** 10.3390/plants14060845

**Published:** 2025-03-08

**Authors:** Xia Chen, Hao Liu, Lanlan Dong, Junrui Shi, Zhonghua Ma, Leiyan Yan, Yanni Yin

**Affiliations:** 1 Key Laboratory of Biology of Crop Pathogens and Insects of Zhejiang Province, Institute of Biotechnology, Zhejiang University, 866 Yuhangtang Road, Hangzhou 310058, China; 22016237@zju.edu.cn (X.C.); 19825008848@163.com (H.L.); 12116085@zju.edu.cn (L.D.); 22116214@zju.edu.cn (J.S.); zhma@zju.edu.cn (Z.M.); 2 Ningbo Academy of Agricultural Sciences, Ningbo 315040, China

**Keywords:** luffa, *Fusarium incarnatum*, leaf spot and fruit rot

## Abstract

In 2022, an outbreak of fungal rot disease affected luffa crops in Shanghai and Zhejiang Province. Infected plants exhibited symptoms including yellowing, chlorosis, wilting, and water-soaked occurred on leaves and fruits. Dark brown, concave lesions developed, often accompanied by white or pale pink mold under moist conditions. Fourteen pathogen strains, morphologically resembling *Fusarium* species, were isolated. Molecular analysis confirmed *Fusarium incarnatum* as the causative agent. Pathogenicity tests on luffa plants fulfilled Koch’s postulates, with inoculated plants displaying the same symptoms. Re-isolation of the fungus from the inoculated plants confirmed its role in the disease. To our knowledge, this is the first report of *F. incarnatum* causing leaf spot and fruit rot on luffa in China. Moreover, the soil bacterial strain *Bacillus velezensis* BV171 displayed strong inhibition of *F. incarnatum* mycelia growth and promoted the growth of sponge gourd plants. These findings lay the foundation for the development of diagnostic tools, disease management strategies, and the breeding of resistant luffa varieties.

## 1. Introduction

Sponge gourd (*Luffa cylindrica*), which belongs to the Cucurbitaceae family, is a nutrient-rich vegetable containing potassium, calcium, magnesium, and vitamin A [1], is an important crop grown throughout China, particularly in the southern and northern regions. It is widely cultivated as a summer and autumn vegetable and has numerous industrial [2] and biotechnological applications [3]. Due to its potential medicinal value [4], various parts of the plant, including the leaves, seeds, and fruits, as well as its extracts, have been used in traditional medicine to treat a range of diseases [5,6,7]. However, during its growth, sponge gourd is highly susceptible to infection by various pathogenic fungi, such as *Fusarium* wilt caused by *Fusarium oxysporum* (Fo) [8], crucial leaf diseases such as anthracnose caused by *Colletotrichum fructicola* and *Colletotrichum siamense* [9], downy mildew and leaf spot, caused by *Pseudoperonospora cubensis* [10] and *Alternaria tenussima* [11], respectively. Additionally, fruit rot diseases, including those caused by *Pythium aphanidermatum* [12], *F. incarnatum*, and *Fusarium chlamydosporum* [13], have severely constrained its production. *Fusarium* species are ubiquitous, adopting intricate infection strategies that exhibit remarkable environmental adaptability and potential pathogenicity. These fungi can infect a wide range of crops, leading to wilts, blights, rots, and cankers [14]. Among the affected crops, cucurbits are particularly vulnerable to *Fusarium* infections [15,16]. Cucurbit crops are often grown under greenhouse conditions with continuous cropping, where the high temperature and humidity create an ideal environment for *Fusarium* growth and reproduction. This leads to increased disease prevalence of *Fusarium* species, stunted plant growth, wilting, and eventual death, resulting in significant yield and quality losses and substantial economic damage. *Fusarium* fruit rot is an important soil-borne fungal disease caused by *Fusarium* spp. In cucurbit crops, *Fusarium oxysporum* and *Fusarium solani* are the primary agents of wilt, fruit rot, and root rot diseases [17,18]. However, there have been no reports of *F. incarnatum* causing luffa rot in China.

In the autumn of 2022, symptoms of yellowing and rot in both leaves and fruits were observed in several luffa (*Luffa cylindrica*) cultivation areas in Shanghai and Zhejiang Province, China. Approximately 30% of the luffa plants in the affected farms showed signs of infection. Initially, the leaves and fruits exhibited yellowing and chlorosis, followed by the development of small brown spots. These spots gradually expanded, forming large lesions, which ultimately led to leaf wilting, desiccation, and fruit blackening and decay. Pathogen isolation and identification indicated that *F. incarnatum* was the primary pathogen of *Luffa cylindrica*. The pathogen was first identified in Egypt in 2024 as a *Luffa cylindrica* rot pathogen [13]. *F. incarnatum* is a well-known crop rot pathogen and has been reported globally, causing fruit rot in Muskmelons [19], cucumber [20], peach [21], pepper [22,23], strawberry [24] and litchi [25], posing a significant threat to crop production. Therefore, it is essential to explore new measures to control *F. incarnatum* diseases. Biological control, which utilizes microorganisms, offers an eco-friendly alternative. *Bacillus* species are a valuable resource for developing microbial agents due to their fungicidal and bactericidal potential. In recent years, *Bacillus* strains have demonstrated significant effectiveness in managing crop diseases caused by various *Fusarium* species. For instance, *Bacillus velezensis* and *Bacillus cabrialesii* have been shown to suppress *Fusarium* root rot caused by *Fusarium oxysporum* and *Fusarium moniliforme* [26]. Additionally, *Bacillus* species have mitigated the negative effects of *Fusarium oxysporum* on banana plants affected by *Fusarium* wilt [27,28]. *Bacillus velezensis* has also reduced *Fusarium* head blight and mycotoxin accumulation [29,30], while *Bacillus cabrialesii* has shown efficacy against *Fusarium languescens*-induced potato diseases [31]. Therefore, screening *Bacillus* strains capable of inhibiting *Fusarium* growth holds promise for developing effective biocontrol agents to combat Fusarium rot in crops. The aim of the present study was to investigate the diseased sponge gourd from Shanghai and Zhejiang Province in order to identify the pathogens impeding the normal growth of this crop and the way of their inhibition.

## 2. Results

### 2.1. Sponge Gourd Rot Symptoms Caused by F. incarnatum

**Figure 1:**
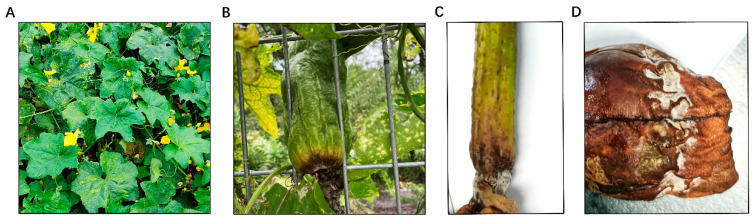
Symptoms of the disease on luffa sponge gourd leaves (**A**) and fruits (**B**–**D**) in the field.

In September 2022, we received samples of diseased sponge gourd leaves and fruits from Shanghai and Zhejiang Province. Our investigation revealed that symptoms mainly occurred in the leaves and fruits of sponge gourd. In the early stages of infection, symptoms included light yellow, irregular chlorosis spots lesions, and wilting of the leaves, followed by the appearance of small, slightly sunken, water-soaked spots (Figure 1A). Similarly, infected areas on the fruits initially turned yellow or developed small brown spots (Figure 1B). As the disease progressed, the lesions turned brown and began to rot, with the centers deepening to a dark brown color and expanding outward, often with a distinct concavity. Under dry conditions, lesion expansion slowed or halted, and no mold formation occurred, exhibiting only yellowing or irregular, spindle-shaped, or circular brown spots (Figure 1C). Conversely, under moist conditions, the lesions spread rapidly, forming a layer of white or pale pink mycelium (Figure 1D).

### 2.2. Fungal Isolation and Morphological Characterization

**Figure 2:**
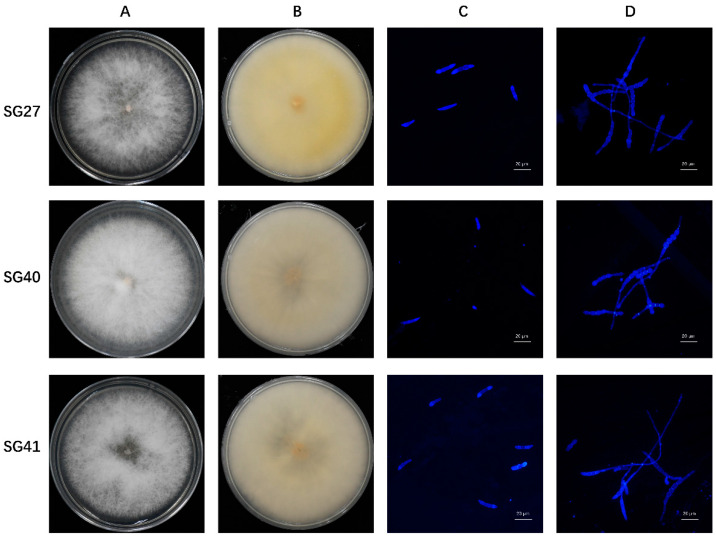
Morphological characteristics of colonies, conidia, and germination of the *Fusarium incarnatum* strains isolated from sponge gourd. Colony morphology of the positive sides (**A**) and reverse sides (**B**) of the *Fusarium incarnatum* strains SG27, SG40, and SG41 on PDA. Microscopic observation of the conidia characteristics (**C**) and germination (**D**). Scale bar = 20 μm.

Leaves and fruit rot can lead to tissue disintegration and plant death, as evidenced by wilting and drying of the leaves and blackening and rotting of the fruits. Fourteen strains with similar morphologies were isolated and purified from the diseased samples and subsequently cultured on a PDA medium. Three strains, which were isolated from sponge gourd plants, were selected for further study. The single-spore isolates formed rounded colonies and produced large amounts of flocculent aerial hyphae. Initially, the hyphae were white, gradually turning beige and pink in the center part of the plate as the culture progressed (Figure 2A,B). The hyphae have transverse septa and branches. The large conidia were sickle-shaped, slightly curved from top to bottom, and tapered at both ends, with 3 to 5 septa, 2.52 to 4.81 × 15.45 to 30.24 µm (3.44 ± 0.53 × 22.97 ± 3.81 µm) (n = 50). The small conidia were ovoid, with 0 to 2 septal, 2.01 to 3.77 × 4.29 to 14.83 µm (2.66 ± 0.44 × 10.61 ± 2.71 µm) (n = 50) (Figure 2C). To further examine the conidial germination, the conidia of each strain were incubated in 2% glucose. After 16 h of incubation, nearly all the conidia had germinated (Figure 2D).

### 2.3. Pathogen Molecular Identification

To further identify the pathogen, the internal transcribed spacer (ITS), transcriptional elongation factor 1-α (EF1-α), and RNA polymerase II second largest subunit (RPB2) were amplified and sequenced from representative isolates SG27, SG40, and SG41, DNA was extracted from 5-day-old aerial mycelium, and PCR was conducted using primers described previously [32]. The obtained nucleotide sequences were deposited in GenBank (ITS: PP507038, PP493955, and PP493956; EF1-α: PP507027, PP507028, and PP507029; and RPB2: PP507033, PP507034, and PP507035). BLASTn search of the sequences revealed 99 to 100% identity with the ITS (MT563420), EF1-α (MN901601), and RPB2 (KF255545) of *Fusarium incarnatum* isolates.

**Figure 3:**
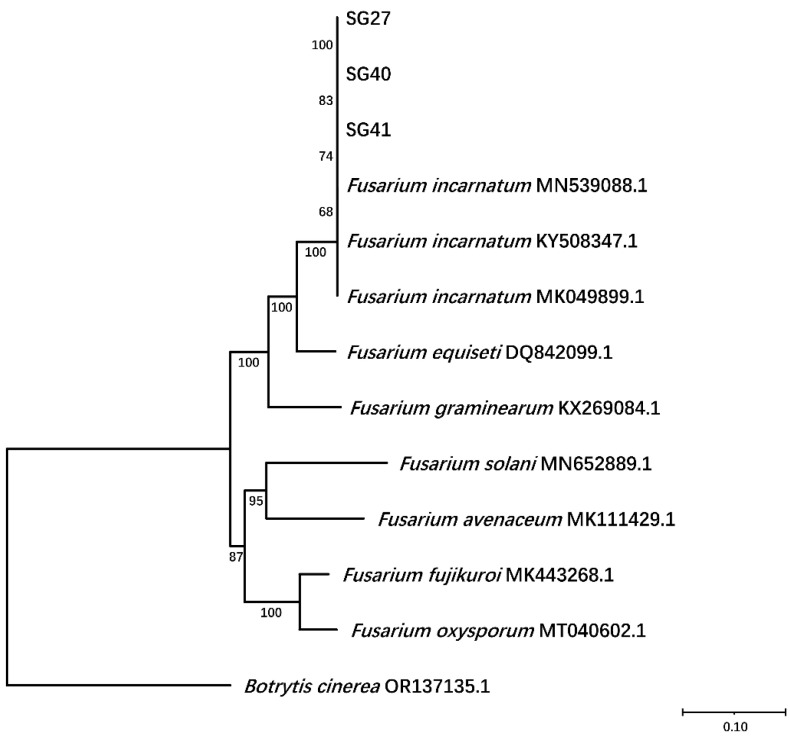
Construction of phylogenetic tree based on the joint concatenated nucleotide sequence of the internal transcribed spacer (ITS), transcriptional elongation factor 1-α (EF1-α), and RNA polymerase II second largest subunit (RPB2) of SG27, SG40, and SG41 strains. Notes in the branches indicate the bootstrap values supporting the branches that were calculated from the bootstrap test of 1000 replicates. The scale bar indicates the number of substitutions at each position.

For further phylogenetic analysis, a maximum likelihood tree was constructed based on ITS, EF1-α, and RPB2 sequences was constructed, which included 9 reference *Fusarium* strains and *Botrytis cinerea*. Phylogenetic analysis revealed that the three isolates formed a clade with *F. incarnatum* (Figure 3).

### 2.4. Pathogenicity Test

**Figure 4:**
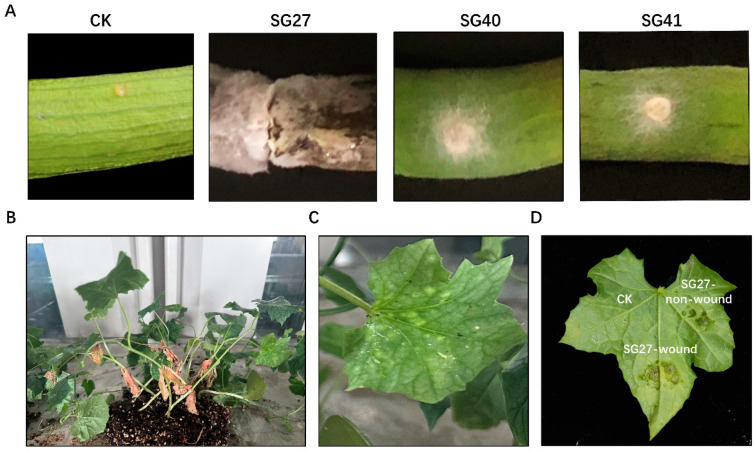
Pathogenicity test of *Fusarium incarnatum* on sponge gourd. (**A**) Symptoms of the sponge gourd fruits caused by SG27, SG40 and SG41. CK indicates the control fruit. (**B**) Symptoms of the sponge gourd plants disease caused by SG27 and an enlarged picture of the diseased leaf (**C**). (**D**) Leaf inoculation with SG27 in vitro.

Firstly, pathogenicity tests were performed by inoculating sponge gourd fruits with mycelial plugs from isolates SG27, SG40, and SG41. Among the three strains, SG27 showed the most severe pathogenicity in fruits, with brown lesions and rot appearing on the fruit surface 6 days after inoculation. No typical symptoms were observed on negative control fruits (Figure 4A). As a result, we selected SG27 as the representative strain responsible for luffa rot disease. To fulfill Koch’s postulates, the pathogenicity tests were conducted by inoculating sponge gourd potted plants with conidial suspensions in vivo. Within 4 days after inoculation, the leaves displayed white lesions, and the surrounding tissue turned yellow and chlorotic, accompanied by the formation of small spots (Figure 4B,C) similar to those observed in the field. In vitro, wounded tissue was more susceptible to infection by *F. incarnatum*, showing leaf spots and rot (Figure 4D). Additionally, the pathogenic fungus was re-isolated from the inoculated plants and identified using the methods described earlier.

### 2.5. Biological Control of F. incarnatum by Bacteria

**Figure 5:**
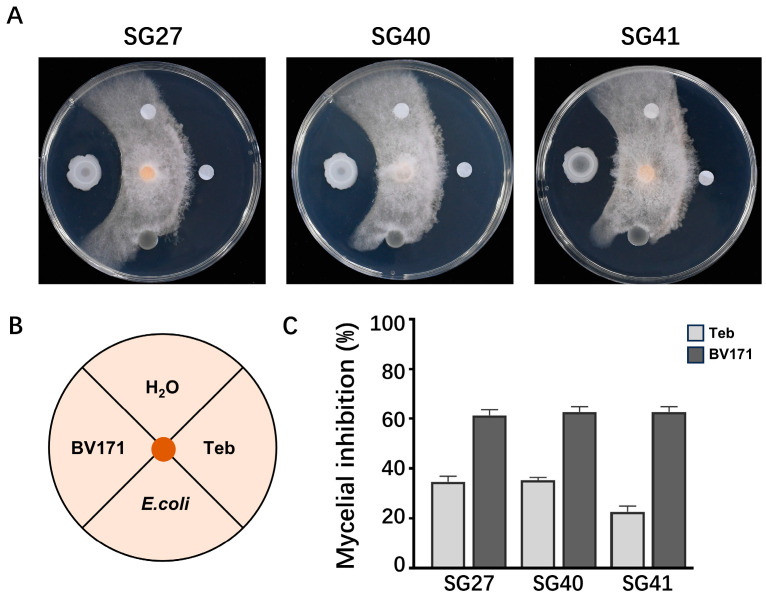
Determination of the biocontrol effect of *Bacillus velezensis* BV171 on *Fusarium incarnatum*. (**A**) Examination of the plate antagonistic effect of 171 on fungal strains SG27, SG40, and SG41. (**B**) Inoculation diagram. (**C**) Mycelial inhibition was calculated after 4-day incubation at 28 °C. The values are the means ± SD (n = 3).

To explore the application of biological control against *F. incarnatum*, the soil bacterial strain *Bacillus velezensis* BV171 was evaluated for its antagonistic activity. On WA plates, the BV171 strain displayed strong inhibition of *F. incarnatum* mycelia growth (Figure 5A–C). 

**Figure 6:**
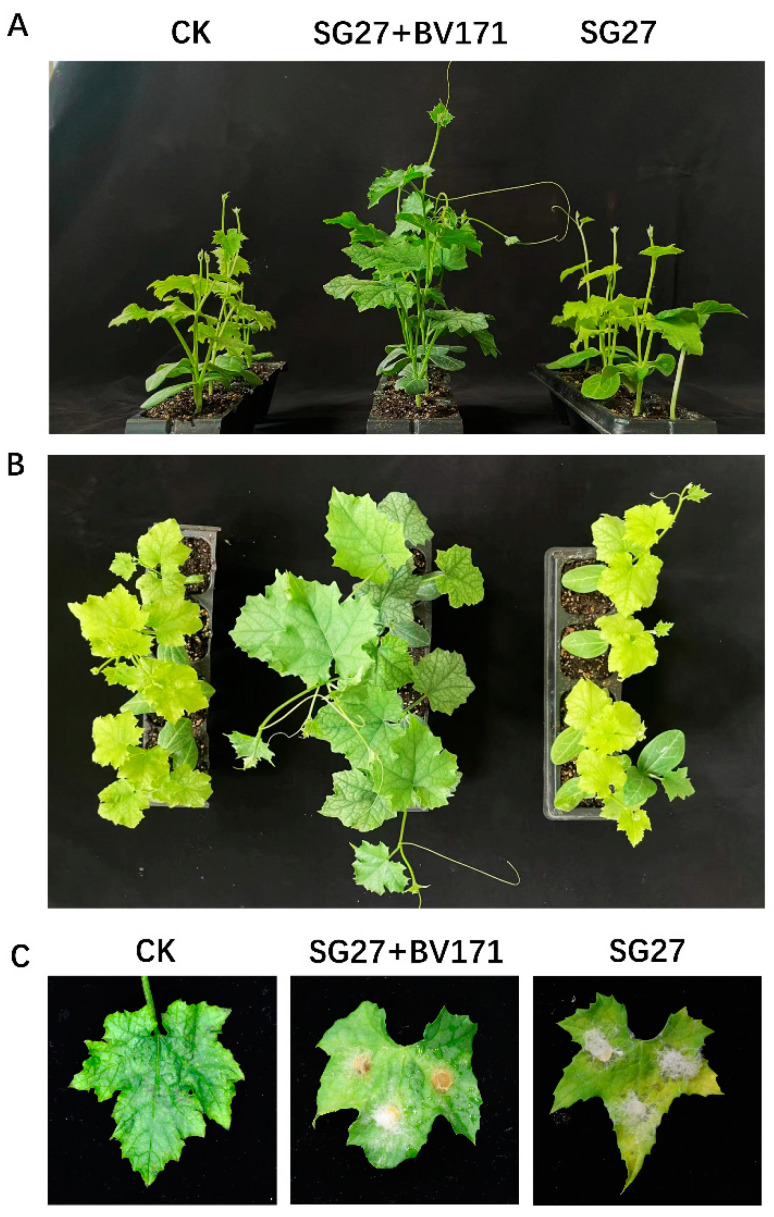
Determination of the biocontrol effect of *Bacillus velezensis* BV171 against *Fusarium incarnatum* on sponge gourd plants. (**A**,**B**) BV171 promotes the growth of sponge gourd plants. (**C**) BV171 can reduce the pathogenicity of *F. incarnatum* on sponge gourd leaves. CK indicates the control without *F. incarnatum* and BV171.

Further, we assessed the biocontrol potential of BV171 against Fusarium rot disease on sponge gourd plants under greenhouse conditions. The results showed that the addition of the BV171 strain significantly promoted the growth of sponge gourd plants (Figure 6A,B) and alleviated the symptoms of Fusarium rot on the leaves (Figure 6C). Our findings suggest that BV171 holds promise as a biocontrol agent for managing Fusarium rot disease in sponge gourd crops.

## 3. Discussion

Previous studies have reported sponge gourd leaf spot and fruit rot caused by *Fusarium oxysporum* [33], *Pseudomonas cichorii* [34], and *Colletotrichum* [9] in China. In this study, we isolated and identified three fungal pathogens from luffa sponge gourd plants with leaf spot and fruit rot symptoms. Through the cultural and microscopic examination, as well as sequence analysis and phylogenetic homology of ITS, TEF-1α, and RPB2, these pathogens were confirmed to be the same species, *Fusarium incarnatum*. These characteristics are consistent with those of *Fusarium incarnatum-equiseti* species complex [32]. Pathogenicity tests revealed that the three strains exhibited different pathogenicity to the sponge gourd, suggesting that further research is needed to explore the pathogenic differentiation among these strains. Notably, SG27 exhibited stronger pathogenicity toward the host (Figure 4A). Based on these findings, we identified this disease in sponge gourd as Fusarium rot. *F. incarnatum* has previously been reported as a pathogen causing leaf spots in cucumbers and melons [16]. To the best of our knowledge, this is the first report of leaf spot and fruit rot on sponge gourd caused by *F. incarnatum* in China.

The *Fusarium* genus is a soil-borne fungus with a broad host range and is an important plant pathogen. It can infect a variety of crops in China, such as rice [35,36], maize [37], wheat [38], vegetables [39,40], fruits [41], etc., causing significant economic losses. In Iran, several research studies have shown that *Fusarium* species causes crown and root rot diseases in wheat [42,43,44]. The distribution of *Fusarium* species is influenced by the surrounding environment, including drought, salinity, and temperature changes. However, luffa diseases caused by *Fusarium* have been rarely reported in China. This study broadens our understanding of plant diseases caused by *F. incarnatum*.

The typical symptom of plants caused by *F. incarnatum* is tissue rot, affecting fruits, stalks, and roots. Numerous studies have reported that *F. incarnatum* can cause diseases in various crops, including rice, maize [45,46], wheat [47], cucumber [48], muskmelon [49], and sugarcane [50] in China. Currently, the fungicidal active ingredients used to control Fusarium rot include Prochloraz, Difenoconazole azoxystrobine, and Natamycin [51]. Additionally, natural antifungal compounds, such as *Sabina chinensis* essential oils [52], have been recommended as potential fungicides due to their strong and long-term antifungal activity against *F. incarnatum*. However, long-term use of fungicides has induced resistant strains to adapt to environmental changes in the field [53], posing a threat to both the environment and human health. Therefore, establishing appropriate management strategies is crucial for controlling *F. incarnatum* disease. Biocontrol using antagonistic microorganisms is considered a promising alternative to synthetic fungicides. At present, *Trichoderma asperellum* T76-14 [19], *Bacillus amyloliquefaciens* B2-5 [54], and Baf1 [55] are known to be strong antagonists of *F. incarnatum*.

To explore the biological control of microbial strain against the Fusarium rot in luffa plants, we isolated an antagonistic strain from luffa soil, identified as *Bacillus velezensis* BV171. Through bacteriostatic assays and pot-based biological control evaluations, our study demonstrates that BV171 effectively inhibits *F. incarnatum* (Figure 5A). Unlike fungicides, which operate through a single mechanism, antagonistic bacteria control plant diseases through multiple mechanisms, making it more difficult for pathogens to develop resistance [56]. Furthermore, the use of antagonistic bacteria is more environmentally friendly and sustainable compared to chemical treatments. Nanotechnology holds immense potential in revolutionizing biological control strategies for crop disease management [57]. The development of innovative, highly effective, and environmentally friendly (green) agents by combining microorganisms with nanomaterials to combat plant pathogens remains an area requiring further research.

## 4. Materials and Methods

### 4.1. Pathogen Isolation and Purification

The diseased luffa plants were collected from cultivation areas in Ningbo, Taizhou, and Jinhua cities in Zhejiang Province, as well as from Fengxian District in Shanghai, China. Lesion tissue from the junction of diseased and healthy plant tissues was excised using a sterile razor blade in a biosafety cabinet. The tissue was then disinfected by soaking in 1% sodium hypochlorite for 3 min, 75% ethanol for 30 s, and subsequently rinsed with sterilized water 3 times. The tissue was cultured on potato dextrose agar (PDA, containing 200 g/L potato, 20 g/L glucose, 1.0% *w/v* agar) medium plate at 25 °C for 3 to 4 days. The colonies were streaked onto PDA plates for purification. Finally, the newly grown colonies were further purified using single-spore isolation.

### 4.2. Pathogen Identification

Molecular biology experiments were conducted to identify the pathogen. Total genomic DNA was extracted using a fungal DNA extraction kit. Mycelia were collected by scraping the surface of colonies grown on PDA plates for 4 days. Gene loci sequences of the nuclear ribosomal internal transcribed spacer (ITS) [58,59], transcriptional elongation factor 1-alpha (TEF-1α) [60], and RNA polymerase II second largest subunit (RPB2) [61,62] from the isolated fungal strains were amplified using the following primers: ITS1/ITS4 for ITS, TEF-lαF/TEF-lαR for TEF-1α, and RPB2-5F2/RPB2-7cR for RPB2.

The PCR reaction system (25 μL) consisted of 12.5 μL Master Mix (Yisheng Biotechnology Co., Ltd., Shanghai, China), 1 μL DNA template, 1 μL each of forward and reverse primers (10 μmol/L), and 9.5 μL ddH2O. The PCR amplification program included an initial denaturation at 94 °C for 5 min, followed by 35 cycles of denaturation at 94 °C for 30 s, annealing at 55 °C for 30 s, and extension at 72 °C for 45 s, with a final extension at 72 °C for 10 min.

The PCR products were checked by electrophoresis on a 1.0% agarose gel in TBE buffer (1×) and observed under UV illumination. The PCR products were then sent to Taingke Biotech (Hangzhou, China) for sequencing. The obtained sequences and reference sequences retrieved from GenBank were aligned using ClustalW in MEGA11. The curated ITS, TEF-lα, and RPB2 sequences were imported into PhyloSuite (v1.2.3) software. Subsequently, the sequences from the three loci (ITS, TEF-lα, RPB2) were concatenated. The optimal evolutionary models and partitioning schemes were selected using PartitionFinder2 (v2.1.1) with the Greedy algorithm and AICc criterion. Maximum likelihood phylogenetic analysis was performed using IQ-TREE (v2.2.0) software to infer the phylogenetic relationships and construct the phylogenetic tree. Finally, the concatenated sequences were uploaded to the NCBI GeneBank database (https://www.ncbi.nlm.nih.gov/) for Blast comparison analysis.

### 4.3. Mycelia Growth, Conidiation and Morphological Characteristics

A 6-mm mycelial plugs from the active colony edges of parental strain were subcultured onto PDA and incubated for 4 days at 25 °C in the dark. For the conidiation assay, 6-mm mycelial plugs of the strain were inoculated in a 50-mL flask containing 20 mL of carboxymethyl cellulose (CMC, including 15 g/L sodium carboxymethyl cellulose, 1 g/L yeast extract, 1 g/L NH_4_NO_3_, 1 g/L KH_2_PO_4_ and 0.5 g/L MgSO_4_·7H_2_O, pH 6.5) liquid medium. The flasks were incubated at 25 °C for 4 days in a shaker at 180 rpm. Subsequently, the number of conidia in each flask was determined using a hemacytometer. The morphology of conidia cultured in CMC liquid medium for 4 days was observed using a Leica TCS SP5 imaging system after staining with the cell wall-damaging agent calcofluor white (CFW) at 10 μg/m. The conidial length was measured with the program image J. Three biological replicates were used for each strain, and each experiment was repeated three times independently.

### 4.4. Pathogenicity Tests of Strains on Sponge Gourd Plants and Fruits

Potted experiments were conducted with wound and non-wound inoculation methods to show if *F. incarnatum* could cause soft rot on Sponge gourd. Healthy leaves were cut from potted plants, and fruits were purchased from the supermarket. Pathogenicity was confirmed by inoculating healthy loofah leaves and fruits with *F. incarnatum* mycelial plugs. Ten leaves and fruits were tested per treatment, and leaves and fruits inoculated with sterile agar plugs were used as controls.

For further pathogenicity testing on sponge gourd plants, in vivo, inoculations were performed on potted sponge gourd plants with conidial suspensions. The strains were cultured in a CMC liquid medium at 28 °C in a shaker incubator (180 rpm/min) for 4 days. The culture was then filtered with 3 layers of lens-wiping paper to harvest the conidial suspensions, which were adjusted to a final concentration of 10^5^ spores/mL by a hemocytometer. Inoculation was carried out by spraying 5 mL of conidial suspension onto each plant. Control plants were misted with sterile distilled water. All inoculated plants were kept in a plastic chamber with 95% relative humidity at 25 °C under a 16/8 h light/dark cycle until disease symptoms appeared. The experiment was repeated three times. After the plants exhibited disease symptoms, the pathogenic fungi were re-isolated, purified, and identified from the diseased tissues. If the re-isolated pathogenic fungi matched the originally inoculated pathogen based on both morphological and molecular characteristics, this would comply with Koch’s postulates.

### 4.5. Determination of Antagonistic Activity

The antagonism of bacterial isolates was tested by assessing their ability to suppress fungal growth. Firstly, bacteria were cultured on a WA plate (WA, including 5 g/L peptone, 10 g/L glucose, 3 g/L beef extract, 5 g/L NaCl, PH 6.7), and the fungi were cultured on PDA at 28 °C. In the culture tests, the antifungal bioassay was performed on WA. Secondly, the bacteria were inoculated onto WA for 24 h. Finally, a 6-mm mycelial plug of the pathogenic fungus, collected from the edge of an actively growing colony, was placed at the center of WA. The bacteria were cultured around the target fungus at a distance of 2.5 cm. Water and non-antifungal bacteria (*E. coli*) were used as negative controls, and triazole pesticide Tebuconazole (Teb), which has activity against *Fusarium*, was used as a positive control. The culture plates were incubated at 28 °C for 4 days.

### 4.6. Data Analysis

Statistical analyses were conducted using GraphPad Prism 8.0. The values are the means ± SD (n = 3). Analysis of variance (ANOVA) on the conidial length and width was performed. Means were compared using the least significant difference test at a significance level of *p* = 0.05.

## 5. Conclusions

This study provides the first identification of *F. incarnatum* as the pathogen of leaf spot and fruit rot in luffa sponge gourd in China. The pathogenic fungi were isolated and identified based on morphological characteristics, phylogenetic analysis, and pathogenicity tests. Additionally, the biocontrol bacterium *Bacillus velezensis* BV171 was found to effectively control the sponge gourd disease caused by *F. incarnatum*, exhibiting significant bioactive properties with promising potential for the development of natural antibacterial agents. These results might provide new insights into the prevention and management of sponge gourd diseases. 

## Data Availability

The data presented in this study are available on request from the corresponding author.
